# Clinical experience with cranial nerve palsy in patients infused with ciltacabtagene autoleucel for the treatment of relapsed/refractory MM in CARTITUDE-1, -2, and -4

**DOI:** 10.1038/s41408-025-01410-w

**Published:** 2025-11-26

**Authors:** Paula Rodríguez-Otero, Surbhi Sidana, Mathilde Kouwenhoven, Jordan M. Schecter, Nikoletta Lendvai, Kevin C. De Braganca, Ana Slaughter, Carolina Lonardi, Philip Vlummens, Helen Varsos, Christina Corsale, Deepu Madduri, Hao Zhao, Katherine Li, Erin Lee, Loreta Marquez, Man Zhao, Tzu-min Yeh, Diana Chen, Vicki Plaks, Rocio Montes de Oca, Erika Florendo, Nitin Patel, Muhammad Akram, Mythili Koneru, Bianca D. Santomasso, Jaime Gállego Perez-Larraya, Niels WCJ van de Donk

**Affiliations:** 1 Cancer Center Clínica Universidad de Navarra, Cima, CIBERONC, Pamplona, Spain; 2 Stanford University School of Medicine, Stanford, CA USA; 3 Amsterdam University Medical Center, Vrije Universiteit Amsterdam, Amsterdam, The Netherlands; 4 Johnson & Johnson, Raritan, NJ USA; 5 Johnson & Johnson, Zug, Switzerland; 6 Johnson & Johnson, Buenos Aires, Argentina; 7 Johnson & Johnson, Beerse, Belgium; 8 Johnson & Johnson, Spring House, PA USA; 9 Johnson & Johnson, Titusville, NJ USA; 10 IQVIA, Shanghai, China; 11 Johnson & Johnson, Shanghai, China; 12 Legend Biotech USA Inc., Somerset, NJ USA; 13 Department of Neurology, Memorial Sloan Kettering Cancer Center, New York, NY USA; 14 Neurology Department, Clínica Universidad de Navarra, CIMA, IDISNA, Pamplona, Spain

**Keywords:** Health care, Medical research

Ciltacabtagene autoleucel (cilta-cel), an approved B-cell maturation antigen (BCMA)-directed CAR T-cell therapy, showed strong efficacy in heavily pre-treated patients with RRMM and in patients at earlier lines of therapy (LOT) in the single-arm CARTITUDE-1, multi-cohort CARTITUDE-2, and phase 3 CARTITUDE-4 trials [1–6]. The cilta-cel adverse event (AE) profile was consistent with that of the CAR-T class, which includes immune-related toxicities like cytokine release syndrome (CRS) and neurologic events like immune effector cell–associated neurotoxicity syndrome (ICANS) [6, 7]. Reports of cranial nerve (CN) palsy (CNP) following CAR T-cell therapy are infrequent [2, 8–12], and guidance on its management is needed. We describe clinical experience with CNP presentation and management after cilta-cel in CARTITUDE-1, CARTITUDE-2 cohorts A, B, and C, and CARTITUDE-4.

Clinical analyses included patients with RRMM in these studies who received cilta-cel as study treatment. Study designs, including AE assessments, were previously reported (Supplementary Methods) [1, 3–6].

Patients who developed CNP underwent diagnostic workup at investigator discretion; pharmacokinetic and biomarker analyses herein were conducted on peripheral blood from patients in CARTITUDE-4 who received cilta-cel (Supplementary Methods).

At data cutoffs, median follow-up was 33.4 months in CARTITUDE-1; 22.9, 27.9, and 26.2 months in CARTITUDE-2 cohorts A, B, and C, respectively; and 15.9 months in CARTITUDE-4. A total of 332 patients received cilta-cel as study treatment. Twenty-one (6.3% [CARTITUDE-1: 3.1%; CARTITUDE-2 cohorts A, B, and C: 5.0%, 5.3%, and 0%, respectively; CARTITUDE-4: 9.1%]) developed CNP, all presenting as peripheral facial nerve (CN VII) palsy characterized by muscle weakness/paralysis on one (unilateral) or both (bilateral) sides of the face (Table 1). An additional CN was impaired in three patients (CN III [oculomotor nerve] in one, after CN VII palsy resolution; CN V [trigeminal nerve] in two, both simultaneous with CN VII palsy). Maximum CNP severity was grade 2 in 18 patients, and grade 3 in three (all with additional impaired CN). Median time to onset was 22 days (range, 17–101) after cilta-cel. Nine (43%) cases were unilateral (five left-sided; two right-sided; two side unspecified) and eight (38%) were bilateral (four [19%] not reported).

**Table 1:** CNP cases by CARTITUDE study.

	Patients Infused With Cilta-cel as Study Treatment, *N*	Patients With CNP, *n* (%)	Maximum CNP Severity, *n* (%)		Cranial Nerve Involvement in Addition to Cranial Nerve VII, *n* (% [Nerve, Grade])
			Grade 2	Grade 3	
CARTITUDE-1	97	3 (3.1)	2 (2.1)	1 (1.0)	1 (1.0 [V, 3])
CARTITUDE-2, cohort A/initial group	20	1 (5.0)	1 (5.0)	0	0
CARTITUDE-2, cohort B	19	1 (5.3)	1 (5.3)	0	0
CARTITUDE-2, cohort C	20	0	–	–	–
CARTITUDE-4	176	16 (9.1)	14 (8.0)	2 (1.1)	2 (1.1 [III, 3; V, 3])
Total	332	21 (6.3)	18 (5.4)	3 (0.9)	3 (0.9)

*Cilta-cel* ciltacabtagene autoleucel, *CNP* cranial nerve palsy.

Thirteen (62%) patients with CNP had ≥1 concurrent neurologic AE (CAR-T related) or concurrent neurologic symptom (not considered CAR-T related by investigator) (Supplementary Results). Headache, typically retro-auricular, was the only concurrent neurologic AE/symptom reported by multiple (seven) patients. No patients with CNP reported Guillain-Barré syndrome or Parkinsonism at any time.

Baseline characteristics were generally comparable in patients with versus without CNP (Table S1), except 81% versus 58% were male and 67% versus 41% had high-risk cytogenetics. Of 232 patients with 1–3 prior LOT and 100 with ≥3 prior LOT, a respective 19 (8%) and two (2%) developed CNP. Baseline characteristics were assessed post hoc for association with CNP in CARTITUDE-4 (Table S2); none were associated with CNP (data not shown).

In patients with versus without CNP, CRS incidence was 91% versus 82% (grade 2, 33% vs 29%; grade ≥3, 0 vs 3%); CRS timing was comparable (Table S3). Tocilizumab was given to 62% versus 51% of patients. CRS resolved in all patients with CNP. ICANS occurred in one patient (grade 2) with CNP and resolved before CNP onset.

Twenty-one (100%) patients with CNP and 303 (97%) without CNP received anti-viral prophylaxis over the first 100 days after cilta-cel. Viral infections occurred after infusion and before CNP onset in two (10%) patients—both cytomegalovirus infections and both treated with ganciclovir and valganciclovir (plus intravenous immunoglobulin in one); 23 (7%) patients without CNP had viral infections during the first 31 post-infusion days (Table S4). Bacterial infections occurred in five patients after cilta-cel and before CNP onset (Supplementary Results); one had both bacterial and cytomegalovirus infections. Two infections (one bacterial [perichondritis]; one cytomegalovirus) were unresolved by CNP onset; both were not assessed by the investigator to have caused CNP.

A CARTITUDE-4 post hoc analysis identified no associations between grade ≥2 CRS, any-grade ICANS, or viral infection during the first 21 post-infusion days and risk of CNP (data not shown).

Brain MRI was conducted in 17 (81%) patients with CNP. Although not all MRIs assessed CN VII, enhancement was observed in 7/21 (33%) cases. Serologic testing for anti-ganglioside antibodies (seen in Guillain-Barré syndrome and variant disorders) was performed for two patients; autoantibodies were undetectable in both. Cerebrospinal fluid (CSF) analyses were conducted in 14 (67%) patients with CNP (Supplementary Results). There was no evidence of active CSF infection, and CSF microscopy and immunophenotypic analyses detected no signs of malignant cells. Together, these tests revealed no signs of tumor infiltration or infection in the central nervous system in any patients analyzed.

Nineteen patients received corticosteroids for CNP (Table S5). The starting dose for the majority was either 10 mg dexamethasone every 6 h or 1 mg/kg prednisolone, with total duration including taper of ≤14 days.

By data cutoff, 19/21 CNP cases resolved, including the three grade 3 cases, three involving multiple CNs, and two untreated with corticosteroids. Both unresolved cases subsequently improved to grade 1. Median time from CNP maximum grade to improvement was 26.5 days (range, 1–96). Among resolved cases, the median CNP duration was 66 days (range, 1–209). The unresolved cases were treated with oral prednisone or prednisolone for 10 and 13 days. CNP duration did not differ between patients who received a total corticosteroid dose of <250 versus ≥250 mg dexamethasone/equivalent or between patients who received corticosteroids for ≤14 versus >14 days (Fig. 1).

**Fig. 1:**
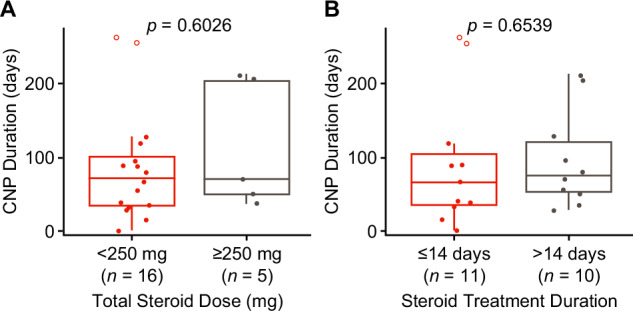
CNP duration by steroid treatment. **A** Patients with total steroid dose of <250 mg versus ≥250 mg of dexamethasone or equivalent while experiencing CNP. **B** Patients with steroid treatment for ≤14 versus >14 days while experiencing CNP. Open circles represent patients with unresolved CNP at clinical cutoff. *CNP* cranial nerve palsy.

Ad hoc correlative analyses of drug product characteristics, CAR-T dose, and immunophenotypes of samples from CARTITUDE-4 are summarized in Supplementary Results. Pharmacokinetics showed significantly higher CAR-T peak expansion and exposure levels in patients with versus without CNP (Fig. S1).

Baseline absolute lymphocyte count (ALC) was comparable in patients with versus without CNP. ALC at time of cilta-cel peak expansion and ALC area under the curve (AUC) from infusion to CNP onset were both higher in patients with CNP (Fig. S2).

Pre-infusion interferon (IFN)–γ levels were significantly lower in patients with versus without CNP; pre-infusion levels of other assessed biomarkers, including serum BCMA, were comparable between the two groups (Figs. S3 and S4). Post infusion, we observed significantly higher peak serum interleukin (IL)–10 levels and trends toward higher peak IL-6 and IL-2Rα levels in patients with versus without CNP, and no differences in peak IFN-γ or ferritin levels. Trends toward higher AUC levels of IL-6 and ferritin were noted in patients with CNP; AUC levels of IL-10 and IL-2Rα were higher in patients with CNP; and no difference in IFN-γ AUC levels was observed (Fig. S5).

In summary, CNP occurred in 6% of patients who received cilta-cel as study treatment. The defining feature was facial nerve (CN VII) palsy; cases involving two CNs were rare. No patients with CNP reported Guillain-Barré syndrome or Parkinsonism. CNP onset was later than the typical onset and resolution of CRS and ICANS. CNP was predominantly low grade and reversible; median duration was 66 days. Most were treated with one oral corticosteroid course, and all cases improved with 19/21 fully resolving. CNP rates were higher after 1–3 versus ≥3 prior LOT, and most cases occurred in males. However, no clinical predictors of CNP were identified in CARTITUDE-4. Overall, incidences of post-infusion viral infections and CRS were comparable between groups, ICANS did not portend CNP development, and no patients reported COVID-19 before CNP.

It is important to rule out tumoral and infectious causes of CNP before initiating corticosteroids. In all cases where performed, brain MRI and CSF analyses showed no sign of infection, tumor infiltration, or tumoral lesions/compression of affected CN(s).

Facial nerve palsy pathogenesis is not fully understood, but may be related to local inflammation in the region where CN VII exits the stylomastoid foramen [13]. Our pharmacokinetic and biomarker data and our observation of higher CNP prevalence at early LOT, when immune fitness may be greater [14], suggest the underlying pathophysiology of CNP after CAR-T is immunologic. This is consistent with the CAR-T class’s mechanism and associated immune-related toxicities. Further research on immunologic mechanisms is needed.

Limitations of our report include differences in diagnostic workup between study sites. CSF analyses were conducted in most but not all patients with CNP. Anti-ganglioside antibody assessments were only performed in two patients; however, these tests were likely to be uninformative because cilta-cel eliminates plasma cells.

The small number of heavily pre-treated patients with CNP in our analysis limits extrapolation of incidence to larger populations. However, rates in CARTITUDE-1 (3%) were generally comparable with real-world rates in heavily pre-treated patients (5%) [15], with differences potentially due to sample size.

In conclusion, patients treated with cilta-cel may experience CNP involving CN VII. After excluding infection and leptomeningeal disease as potential causes, we propose treating patients with a short, low-dose corticosteroid course (standard for Bell’s palsy), as we observed no difference in CNP duration based on corticosteroid dose or duration, and it is important to avoid excessively high corticosteroid doses and long-term use. Most cases resolved and were treated with one corticosteroid course.

## Supplementary information

Supplementary (PDF)

## Data Availability

The data sharing policy of Janssen Pharmaceutical Companies of Johnson & Johnson is available at https://www.janssen.com/clinical-trials/transparency. As noted on this site, requests for access to the study data can be submitted through Yale Open Data Access (YODA) Project site at http://yoda.yale.edu.
