# Supplementary material for: Clinical experience with cranial nerve palsy in patients infused with ciltacabtagene autoleucel for the treatment of relapsed/refractory MM in CARTITUDE-1, -2, and -4
Source: Blood Cancer J. 2025 Nov 26;15(1):209. doi: 10.1038/s41408-025-01410-w (PMC12658040; doi:10.1038/s41408-025-01410-w)
Supplement: Supplementary file 1 — Supplementary (PDF) [file 41408_2025_1410_MOESM1_ESM.pdf]

Clinical experience with cranial nerve palsy in patients infused with ciltacabtagene autoleucel for the treatment of relapsed/refractory MM in CARTITUDE-1, -2, and -4

Paula Rodríguez-Otero; Surbhi Sidana; Mathilde Kouwenhoven; Jordan M. Schecter; Nikoletta Lendvai; Kevin C. De Braganca; Ana Slaughter; Carolina Lonardi; Philip Vlummens; Helen Varsos; Christina Corsale; Deepu Madduri; Hao Zhao; Katherine Li; Erin Lee; Loreta Marquez; Man Zhao; Tzu-min Yeh; Diana Chen; Vicki Plaks; Rocio Montes de Oca; Erika Florendo; Nitin Patel; Muhammad Akram; Mythili Koneru; Bianca D. Santomasso; Jaime Gállego Perez-Larraya; Niels WCJ van de Donk

Table of Contents

Supplementary Methods **3**

Supplementary Results **5**

Supplementary References **7**

Table S1 Baseline characteristics in patients with and without CNP in CARTITUDE-1, CARTITUDE-2, cohorts A, B, and C, and CARTITUDE-4 .... **9**

Table S2 List of baseline and early post-baseline parameters assessed post hoc for association with CNP in CARTITUDE-4 .... **11**

Table S3 CRS in patients with and without CNP in CARTITUDE-1, CARTITUDE-2, cohorts A, B, and C, and CARTITUDE-4 .... **12**

Table S4 Post–cilta-cel, pre-CNP viral infections and antiviral prophylaxis **13**

Table S5 List of corticosteroid treatments while CNP was ongoing........**14**

Figure S1 CAR+ T-cell pharmacokinetics in patients with and without CNP in CARTITUDE-4. (A) C_{max} and (B) $AUC_{0\text{-CNP onset}}$........**16**

Figure S2 ALC levels in patients with and without CNP in CARTITUDE-4. (A) ALC levels at baseline, (B) ALC levels at time of cilta-cel C_{max}, and (C) $AUC_{0\text{-CNP onset}}$ of ALC levels **17**

CNP in CARTITUDE-1, -2, and -4

Figure S3 Pre-infusion levels of serum inflammatory markers in patients with and without CNP in CARTITUDE-4. (A) IL-6, (B) IL-10, (C) IL-2Rα, (D) IFN-γ, and (E) ferritin **18**

Figure S4 Pre-infusion serum BCMA levels in patients with and without CNP in CARTITUDE-4 .... **19**

Figure S5 Post-infusion levels of serum inflammatory markers in patients with and without CNP in CARTITUDE-4. C_{max} of (A) IL-6, (B) IL-10, (C) IL-2Rα, (D) IFN-γ, and (E) ferritin. $AUC_{0\text{-CNP onset}}$ of (F) IL-6, (G) IL-10, (H) IL-2Rα, (I) IFN-γ, and (J) ferritin **20**

Figure S6 (A) CD4:CD8 ratio in DP, (B) CAR+ viable T-cells/kg in DP, (C) VCN/transduced cell in DP, and (D) CAR+ T-cell dose and in patients with and without CNP in CARTITUDE-4 .... **21**

Figure S7 (A) T_{max} and (B) T_{last} in patients with and without CNP in CARTITUDE-4 .... **23**

Figure S8 Memory T-cell phenotypes at T_{max} in patients with and without CNP in CARTITUDE-4. (A)CAR+ CD4+ T-cell compartment and (B) CAR+ CD8+ T-cell compartment......................... **24**

CNP in CARTITUDE-1, -2, and -4

Supplementary Methods

Study design and treatment

CARTITUDE-1 (phase Ib/II) assessed safety and efficacy of ciltacabtagene autoleucel (cilta-cel) [1]. Eligible patients had at least three prior lines of therapy (LOT), including a proteasome inhibitor (PI), immunomodulatory drug (IMiD), and anti-CD38 antibody, or were double-refractory to a PI and IMiD and were anti-CD38 antibody exposed.

CARTITUDE-2 is an ongoing, phase II, multi-cohort, open-label study. Cohort A enrolled patients with lenalidomide-refractory relapsed/refractory multiple myeloma after one to three LOT [2]. Cohort B enrolled patients with early relapse (≤ 12 months after frontline therapy/frontline autologous stem cell transplant) [3]. Cohort C enrolled patients with prior PI, IMiD, anti-CD38 antibody, and B-cell maturation antigen (BCMA)-targeting therapy [4].

CARTITUDE-4 is an ongoing, randomized, open-label, phase III study comparing cilta-cel with standard of care [5]. Eligible patients were lenalidomide refractory and had one to three prior LOT, including a PI and IMiD.

In CARTITUDE-1 and CARTITUDE-2, patients received bridging therapy as needed; in CARTITUDE-4, all were to receive ≥ 1 cycle of daratumumab-pomalidomide-dexamethasone or pomalidomide-bortezomib-dexamethasone bridging therapy (physician's choice). In all three studies, patients received one cilta-cel infusion (target dose, 0.75×10^6 chimeric antigen receptor [CAR]+ viable T cells/kg) 5–7 days after start of lymphodepletion (cyclophosphamide 300 mg/m² and fludarabine 30 mg/m² daily for 3 days).

The trials were conducted in accordance with the Declaration of Helsinki and International Council for Harmonisation Guidelines for Good Clinical Practice. All patients provided written informed consent. The independent ethics committee or institutional review board at each site approved the trial protocol.

Safety assessments

In CARTITUDE-4, CARTITUDE-2, and CARTITUDE-1 phase II, cytokine release syndrome (CRS) and immune effector cell–associated neurotoxicity syndrome were graded per American Society for Transplantation and Cellular Therapy consensus criteria [6]; in CARTITUDE-1 phase Ib, CRS was graded per Lee criteria, and investigator-assessed neurotoxicity per Common Terminology Criteria for Adverse Events of the National Cancer Institute, version 5.0 [7,8]. In each study, other adverse events were graded per Common Terminology Criteria for Adverse Events of the National Cancer Institute, version 5.0.

Diagnostic workup

Brain magnetic resonance imaging, lumbar puncture, and assessment of serum anti-ganglioside antibodies were performed in some patients. Electrophysiological studies were not performed. Cerebrospinal fluid (CSF) analyses included chemical analysis (glucose, lactate/lactate dehydrogenase, and protein levels), cytology and immunophenotypic analysis to identify malignant cells and quantify immune cells, and infectious pathogen assessments. Methods to detect infection included nucleic acid amplification tests, microscopy, microbiologic culture, and enzyme-linked immunosorbent assays.

Pharmacokinetic and biomarker analysis

Peripheral blood samples from patients in CARTITUDE-4 who received cilta-cel as study treatment were used to perform pharmacokinetic and biomarker analyses. Analyses were also conducted for patients from CARTITUDE-1 and CARTITUDE-2 (data not shown). CAR+ T-cell levels and T-cell phenotypes in peripheral blood were assessed by flow cytometry. Biomarker levels were assessed by Meso Scale Discovery (Rockville, MD, USA) prior to lymphodepletion, pre infusion on the day of cilta-cel infusion (except for ferritin and absolute lymphocyte count in one patient, for whom samples were collected 2 days before cilta-cel infusion), 1 day prior to

infusion, and post infusion on days 3, 7, 10, 14, 21, 28, 56, 84, and 112. An electrochemiluminescent ligand-binding immunoassay (Johnson & Johnson, Spring House, PA, USA or Malvern, PA, USA) was used to quantify serum BCMA.

Statistical analysis

Data were summarized with descriptive statistics. p values for comparisons of CAR+ T-cell and biomarker levels were determined using the Wilcoxon rank sum test and were intended for descriptive purposes only and unadjusted for multiplicity.

Supplementary Results

Neurologic adverse events and neurologic symptoms concurrent with cranial nerve palsy (CNP)

Concurrent neurologic adverse events and neurologic symptoms that were each reported by one patient were dysgeusia and/or parosmia (both potentially related to cranial nerve VII palsy), restlessness, peripheral sensory neuropathy, polyneuropathy, amnesia, aphasia, agitation, depressed level of consciousness, and diplopia (likely related to cranial nerve III palsy).

Bacterial infections occurring after infusion and before CNP onset

The bacterial infections reported in five patients after cilta-cel and before CNP onset were catheter- or device-related infections, pneumonia, perichondritis, and/or urinary tract infection.

CSF analyses

Glucose levels, total protein levels, and cell counts in CSF were each reported in ten patients. Glucose levels were normal in six and slightly elevated (82–85 mg/dL) or high (90–94 mg/dL) in four. Protein levels were normal in six patients (30–59 mg/dL) and elevated in four (65–101 mg/dL). For seven patients, both CSF white blood cell count and CSF protein level data were reported. White blood cell count was normal in all seven patients, and there was evidence of albuminocytologic dissociation in three patients with elevated protein levels. CSF lactate

dehydrogenase levels were reported in one patient and were normal (26 units/L); lactate levels were reported in two and were normal to elevated (2.0–3.1 mmol/L).

CSF was tested for specific viruses in ten patients. CSF viral panels commonly included cytomegalovirus, enterovirus, Epstein-Barr virus, herpes simplex viruses 1 and 2, human herpesvirus 6, John Cunningham virus (JCV), human parechovirus, and varicella-zoster virus. Bacterial infection assays were conducted for ten patients, including tests for *Borrelia burgdorferi* (Lyme disease) in four and *Treponema pallidum* (syphilis) in two. Six were tested for fungal infection (*Cryptococcus neoformans* and *Cryptococcus gattii*) and three for protozoan infection (*Toxoplasma gondii*). All tests were negative for active CSF infection.

CNP recovery status

By data cutoff, 19 of the 21 CNP cases had fully resolved, including the three grade 3 cases, the three cases involving multiple cranial nerves, and the two cases that were not treated with corticosteroids. Only one patient had relapsing CNP; this patient received COVID vaccination between the first and second CNP episodes and subsequently developed polyradiculoneuritis. With respect to the two unresolved CNP cases, both improved to grade 1 after data cutoff.

Cellular and molecular analyses in CARTITUDE-4

In CARTITUDE-4, the drug product CD4:CD8 ratio was significantly higher in patients with versus without CNP (p = 0.002); other drug product characteristics, as well as CAR+ T-cell dose, were comparable between groups (Figure S6).

There was no difference between groups in CAR+ T-cell persistence (T_{last}; p = 0.402) or time to maximal CAR+ T-cell concentration (T_{max}; p = 0.24), and CAR+ T-cell immunophenotypes were comparable in patients with versus without CNP, with a predominantly central memory phenotype at ~T_{max} (Figures S7–S8).

Supplementary References

1. Berdeja JG, Madduri D, Usmani SZ, Jakubowiak A, Agha M, Cohen AD, et al. Ciltacabtagene autoleucel, a B-cell maturation antigen-directed chimeric antigen receptor T-cell therapy in patients with relapsed or refractory multiple myeloma (CARTITUDE-1): A phase 1b/2 open-label study. Lancet. 2021;398:314–324.

2. Einsele H, Cohen AD, Delforge M, Hillengass J, Goldschmidt H, Weisel K, et al. Biological correlative analyses and updated clinical data of ciltacabtagene autoleucel (cilta-cel), a BCMA-directed CAR-T cell therapy, in lenalidomide (len)-refractory patients (pts) with progressive multiple myeloma (MM) after 1–3 prior lines of therapy (LOT): CARTITUDE-2, cohort A. J Clin Oncol. 2022;40:8020.

3. Van de Donk NWCJ, Agha M, Cohen AD, Cohen YC, Anguille S, Kerre T, et al. Ciltacabtagene autoleucel (cilta-cel), a BCMA-directed CAR-T cell therapy, in patients with multiple myeloma (MM) and early relapse after initial therapy: CARTITUDE-2 cohort B 18-month follow-up. Blood. 2022;140(Suppl 1):7536–7537.

4. Cohen AD, Mateos M-V, Cohen YC, Rodríguez-Otero P, Pavia B, van de Donk NWCJ, et al. Efficacy and safety of cilta-cel in patients with progressive multiple myeloma after exposure to other BCMA-targeting agents. Blood. 2023;141:219–230.

5. San-Miguel J, Dhakal B, Yong K, Spencer A, Anguille S, Mateos M-V, et al. Cilta-cel or standard care in lenalidomide-refractory multiple myeloma. N Engl J Med. 2023;389:335–347.

6. Lee DW, Santomasso BD, Locke FL, Ghobadi A, Turtle CJ, Brudno JN, et al. ASTCT consensus grading for cytokine release syndrome and neurologic toxicity associated with immune effector cells. Biol Blood Marrow Transplant. 2019;25:625–638.

7. Lee DW, Gardner R, Porter DL, Louis CU, Ahmed N, Jensen M, et al. Current concepts in the diagnosis and management of cytokine release syndrome. Blood. 2014;124:188-195.

8. U.S. Department of Health and Human Services: Common terminology criteria for adverse events (CTCAE). Version 5.0. Washington, DC, 2017.

Table S1. Baseline characteristics in patients with and without CNP in CARTITUDE-1, CARTITUDE-2, cohorts A, B, and C, and CARTITUDE-4.

	**CNP (*n* = 21)**	**No CNP (*n* = 311)**
Age, years, median (range)	64 (45–75)	60 (27–81)
Male, *n* (%)	17 (81.0)	180 (57.9)
Time since diagnosis, years, median (range)	3.63 (1.5–9.2)	4.1 (0.3–18.2)
Prior LOT, *n* (%)		
1–3	19 (90.5)	213 (68.5)
1	7 (33.3)	75 (24.1)
2–3	12 (57.1)	138 (44.4)
> 3	2 (9.5)	98 (31.5)
Multiple myeloma type, *n* (%)		
IgA	2 (9.5)	42 (13.5)
IgG	11 (52.4)	168 (54.0)
Light chain	7 (33.3)	77 (24.8)
ISS stage, *n* (%)		
I	19 (90.5)	196 (63.0)
II	1 (4.8)	81 (26.0)
III	1 (4.8)	34 (10.9)
Plasmacytomas, *n* (%)[a]	4 (19.0)	56 (18.0)
Cytogenetics, *n* (%)[b]		
Standard risk	6 (28.6)	148 (47.6)
High risk[c]	14 (66.7)	128 (41.2)
del(17p)	8 (38.1)	58 (18.6)
t(4;14)	1 (4.8)	27 (8.7)
t(14;16)	2 (9.5)	9 (2.9)
Gain/amp(1q)	7 (33.3)	70 (22.5)
Unknown/not reported	1 (4.8)	10 (3.2)
LDH on/prior to lymphodepletion, U/L, median (range)	190 (120–386)	183 (87–619)

Abbreviations: *CNP* cranial nerve palsy, *Ig* immunoglobulin, *ISS* International Staging System, *LDH* lactate dehydrogenase, *LOT* line of therapy.

[a]Assessments included bone-based and extramedullary plasmacytomas in CARTITUDE-1; assessments included soft tissue plasmacytomas in CARTITUDE-2 and CARTITUDE-4.

[b]Cytogenetic risk was assessed in 21 patients with CNP and 287 patients without CNP.

[c]In CARTITUDE-1, any of the following: del(17p), t(4;14), t(14;16). In CARTITUDE-2 and CARTITUDE-4, any of the following: del(17p), t(4;14), t(14;16), gain/amp(1q).

Table S2. List of baseline and early post-baseline parameters assessed post hoc for association with CNP in CARTITUDE-4.

Characteristic
Number of lines of prior therapy
Tumor burden (low, intermediate, high)
Percent bone marrow plasma cells
Disease tempo (percent change in disease burden from screening to baseline)
Duration-adjusted disease tempo (disease tempo per week)
Presence of extramedullary plasmacytomas
Presence of soft tissue plasmacytoma
Cytogenetics high-risk abnormality: del(17p)
Cytogenetics high-risk abnormality: t(4;14)
At least two high-risk cytogenetic abnormalities
Refractory to anti-CD38 monoclonal antibody
Triple-class refractory (PI + IMiD + anti-CD38)
International Staging System stage (I, II, III),
Investigator choice of PVd or DPd
Country
Region
Grade ≥ 2 CRS
Any-grade ICANS
Documented viral infection within the first 21 days after cilta-cel infusion
Ongoing or medical history of neuropathy at time of study entry
New neuropathy with onset between randomisation and 21 days after cilta-cel infusion

Abbreviations: *CNP* cranial nerve palsy, *CRS* cytokine release syndrome, *DPd* daratumumab, pomalidomide, and dexamethasone *ICANS* immune effector cell–associated neurotoxicity syndrome, *IMiD* immunomodulatory drug, *PI* proteasome inhibitor, *PVd* pomalidomide, bortezomib, and dexamethasone.

Table S3. CRS in patients with and without CNP in CARTITUDE-1, CARTITUDE-2, cohorts A, B, and C, and CARTITUDE-4.

	**CNP (*n* = 21)**	**No CNP (*n* = 311)**
CRS events, *n* (%)	19 (90.5)	254 (81.7)
Grade 1	12 (57.1)	154 (49.5)
Grade 2	7 (33.3)	90 (28.9)
Grade 3	0	6 (1.9)
Grade 4	0	3 (1.0)
Grade 5	0	1 (0.3)
Post-infusion time to onset, days, median (range)	7 (3–10)	7 (1–23)
Duration, days, median (range)	3 (1–11)	4 (1–97)
Supportive treatments, *n* (%)	19 (90.5)	247 (79.4)
IL-6 pathway modulators	13 (61.9)	160 (51.4)
Antagonist (tocilizumab)	13 (61.9)	160 (51.4)
Neutralizer (siltuximab)	0	1 (0.3)
Anakinra	1 (4.8)	21 (6.8)
Corticosteroids	4 (19.0)	41 (13.2)
IV fluids	6 (28.6)	61 (19.6)
Vasopressors	0	8 (2.6)
Oxygen	1 (4.8)	27 (8.7)
Analgesics/anti-inflammatory	15 (71.4)	185 (59.5)
Anti-infectives	14 (66.7)	150 (48.2)
Anti-epileptics	0	2 (0.6)
Other	2 (9.5)	22 (7.1)
CRS recovered/resolved, *n/N* (%)[a]	19/19 (100)	251/254 (98.8)

Abbreviations: *CNP* cranial nerve palsy, *CRS* cytokine release syndrome, *IL* interleukin, *IV* intravenous.

[a]Denominator based on number of patients with CRS.

Table S4. Post–cilta-cel, pre-CNP viral infections and antiviral prophylaxis.

	**CNP (*n* = 21)**	**No CNP (*n* = 311)**
Viral infection, *n* (%)	2 (9.5)[a]	23 (7.4)[b]
Cytomegalovirus infection[c]	2 (9.5)[a]	5 (1.6)
Rhinovirus infection	0	5 (1.6)
COVID-19[d]	0	7 (2.3)
Parainfluenza virus infection	0	2 (0.6)
Adenovirus infection	0	1 (0.3)
Bronchiolitis	0	1 (0.3)
Coronavirus infection[e]	0	1 (0.3)
Herpes simplex reactivation	0	1 (0.3)
Metapneumovirus infection	0	1 (0.3)
Viral urinary tract infection	0	1 (0.3)
Viral upper respiratory tract infection	0	1 (0.3)
Antiviral prophylaxis, *n* (%)[f]	21 (100)	303 (97.4)
Acyclovir	17 (81.0)	242 (77.8)
Valaciclovir	6 (28.6)	65 (20.9)
Entecavir	1 (4.8)	3 (1.0)
Lamivudine	0	3 (1.0)
Oseltamivir	0	3 (1.0)
Valganciclovir	0	2 (0.6)
Famciclovir	0	1 (0.3)

Abbreviations: *CNP* cranial nerve palsy.
[a]One case ongoing at time of CNP onset.
[b]Within 31 days after cilta-cel infusion.
[c]Includes cytomegalovirus infection, cytomegalovirus infection reactivation, and cytomegalovirus syndrome.
[d]Includes COVID-19, COVID-19 pneumonia, and asymptomatic COVID-19; does not include “coronavirus” infection.
[e]Does not include COVID-19, COVID-19 pneumonia, or asymptomatic COVID-19.
[f]Received during the first 100 days after infusion.

Table S5. List of corticosteroid treatments while CNP was ongoing.

Case	Maximum CNP Severity	Treatment (Route of Administration)	Duration of Corticosteroid Treatment (Number of Courses)	Total Steroid Dose, mg[a]	CNP Onset, Days After Infusion (Duration)
**CARTITUDE-1**					
Case 1	Grade 2	Dexamethasone (oral)	1 days (1)	200	Day 101 (1 day)
Case 2	Grade 2	Methylprednisolone (oral)<br>Dexamethasone (oral)<br>Prednisone (oral)	52 days (1)	546	Day 26 (70 days)
Case 3	Grade 3	Dexamethasone (IV)<br>Prednisone (oral)	23 days (1)	198	Day 21 (79 days)
**CARTITUDE-2**					
Case 1 (cohort A)	Grade 2	Dexamethasone (oral)	29 days (1)	296	Day 29 (51 days)
Case 2 (cohort B)	Grade 2	Prednisolone (oral)	28 days (2)	165	Day 22 (128 days)
**CARTITUDE-4**					
Case 1	Grade 2	Prednisolone (oral)	46 days (1)	169	Day 21 (95 days)
Case 2	Grade 2	Dexamethasone (IV)	12 days (1)	390	Day 19 (38 days)
Case 3	Grade 2	–	–	–	Day 18 (39 days)
Case 4	Grade 2	Prednisone (oral)	15 days (1)	107	Day 23 (34 days)
Case 5	Grade 3	Dexamethasone (IV)<br>Prednisone (oral)<br>Hydrocortisone (oral)	93 days (2)	652	Day 21 (204 days)
Case 6	Grade 2	Prednisolone (oral)	27 days (1)	183	Day 17 (28 days)
Case 7	Grade 2	Prednisolone (oral)	13 days (1)	71	Day 30 (> 253 days; not resolved)[b]
Case 8	Grade 2	Prednisone (oral)	13 days (1)	66	Day 24 (88 days)
Case 9	Grade 2	Prednisone (oral)	199 days (1)	950	Day 60 (209 days)

		Dexamethasone (IV)<br>Methylprednisolone (oral)			
Case 10	Grade 3	Prednisolone (oral)	46 days (2)	191	Day 22 (56 days)
Case 11	Grade 2	–	–	–	Day 25 (32 days)
Case 12	Grade 2	Prednisone (oral)<br>Hydrocortisone sodium succinate (oral)	11 days (2)	52	Day 25 (118 days)
Case 13	Grade 2	Dexamethasone (IV)<br>Prednisone (oral)	11 days (1)	167	Day 20 (66 days)
Case 14	Grade 2	Prednisone (oral)	6 days (2)	54	Day 21 (89 days)
Case 15	Grade 2	Prednisone (oral)	10 days (1)	78	Day 21 (> 262 days; not resolved)[b]
Case 16	Grade 2	Dexamethasone (oral)	6 days (1)	60	Day 20 (15 days)

Abbreviations: *CNP* cranial nerve palsy, *IV* intravenous.
[a]Total corticosteroid doses are in dexamethasone or equivalent doses (conversion factor = 190). [b]CNP improved to grade 1 after data cutoff.

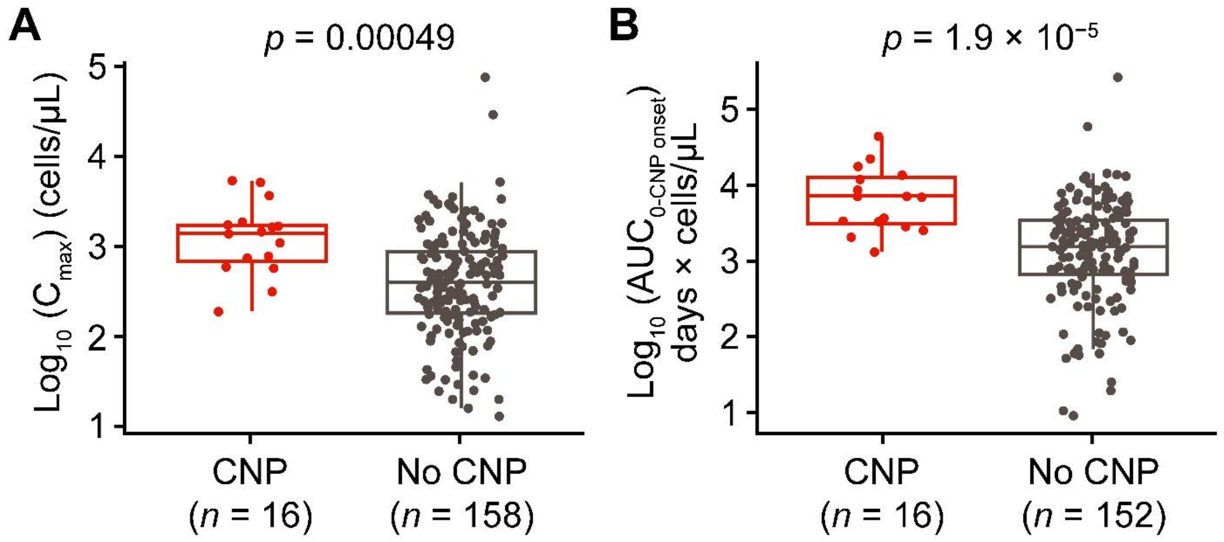

Figure S1. CAR+ T-cell pharmacokinetics in patients with and without CNP in CARTITUDE-4. (A) C_{max} and (B) $AUC_{0\text{-CNP onset}}$. The median days to CNP onset was used to calculate exposure levels at an equivalent timepoint for patients without CNP. *$AUC_{0\text{-CNP onset}}$* area under the concentration-time curve from time 0 to CNP onset, *CAR* chimeric antigen receptor, *C_{max}* maximal level, *CNP* cranial nerve palsy.

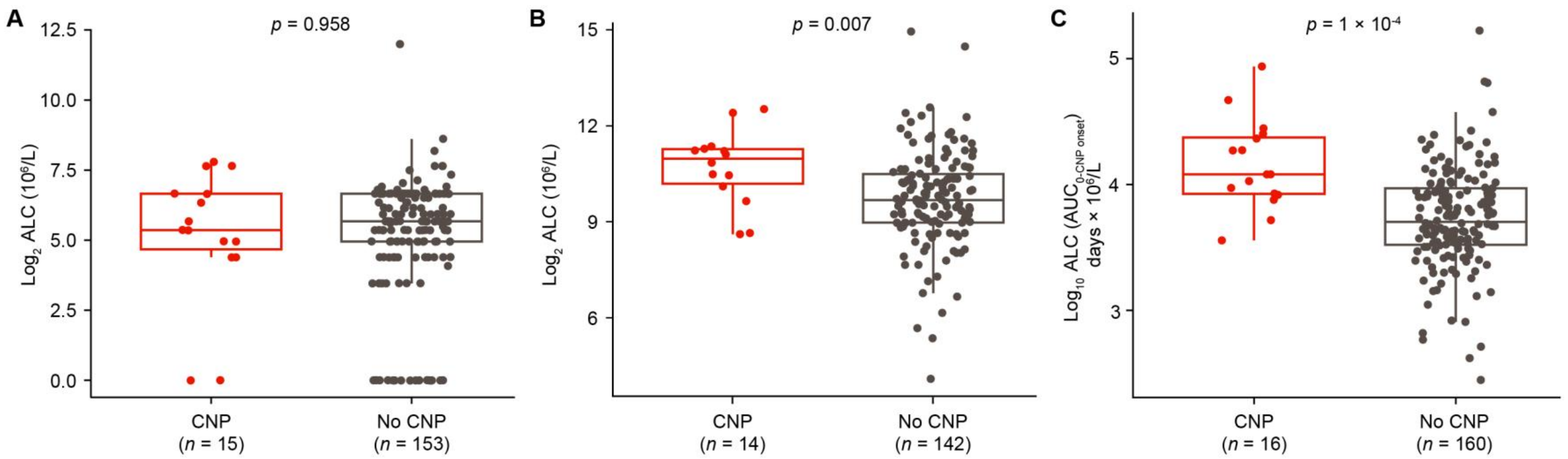

Figure S2. ALC levels in patients with and without CNP in CARTITUDE-4. (A) ALC levels at baseline, (B) ALC levels at time of cilta-cel C_{max}, and (C) $AUC_{0\text{-CNP onset}}$ of ALC levels. The median days to CNP onset was used to calculate an equivalent timepoint for patients without CNP. *ALC* absolute lymphocyte count, $AUC_{0\text{-CNP onset}}$ area under the concentration-time curve from time 0 to CNP onset or an equivalent timepoint, C_{max} maximal level, *CNP* cranial nerve palsy.

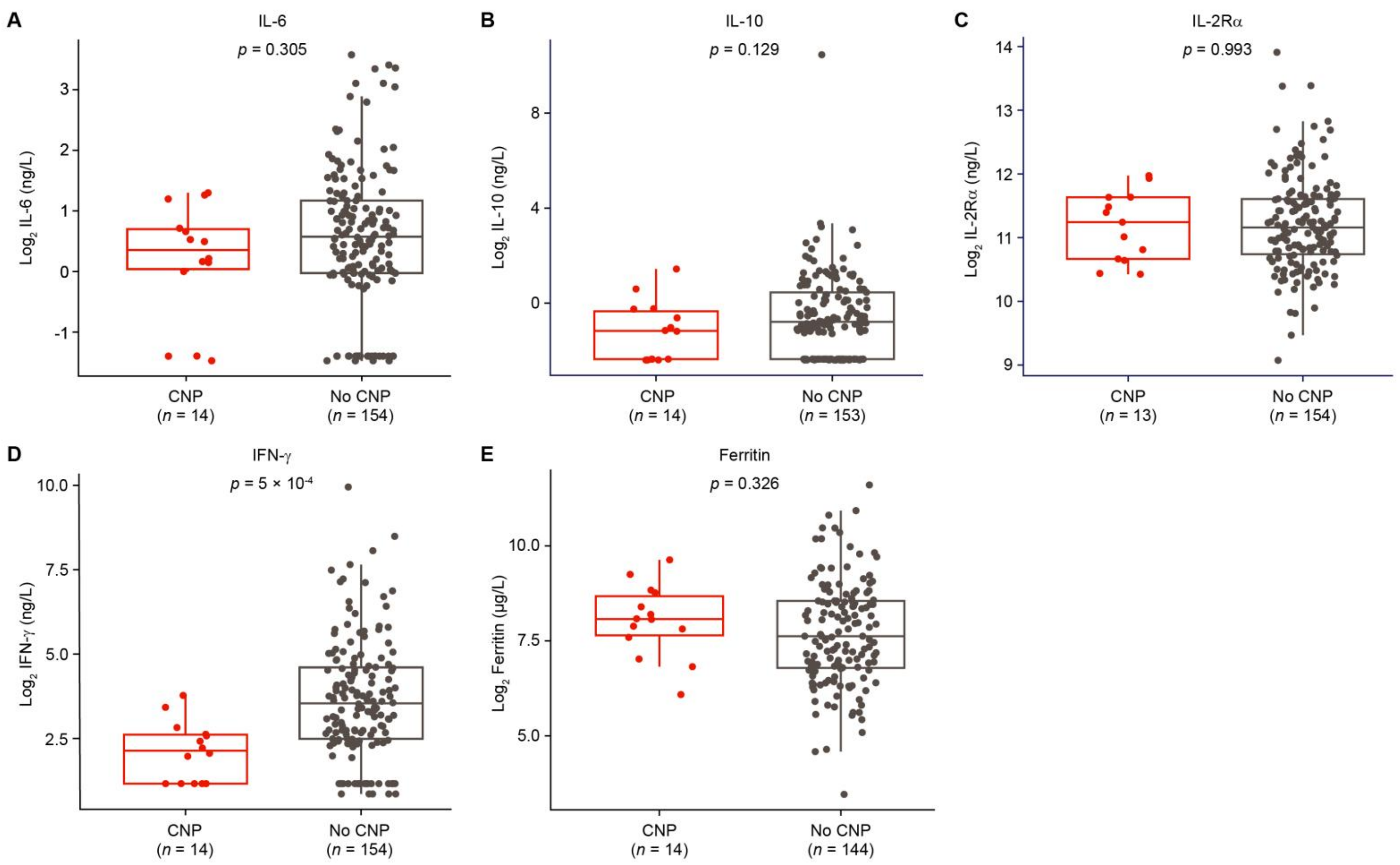

Figure S3. Pre-infusion levels of serum inflammatory markers in patients with and without CNP in CARTITUDE-4. (A) IL-6, (B) IL-10, (C) IL-2Rα, (D) IFN-γ, and (E) ferritin. C_{max} maximal level; *CNP* cranial nerve palsy, *IFN* interferon, *IL* interleukin.

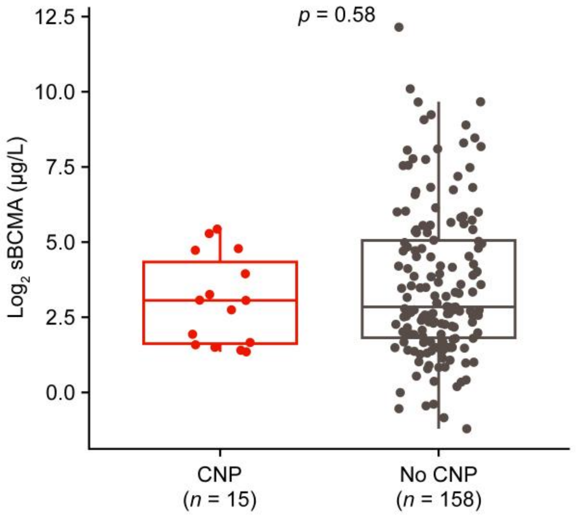

Figure S4. Pre-infusion sBCMA levels in patients with and without CNP in CARTITUDE-4. *CNP* cranial nerve palsy, *sBCMA* soluble B-cell maturation antigen.

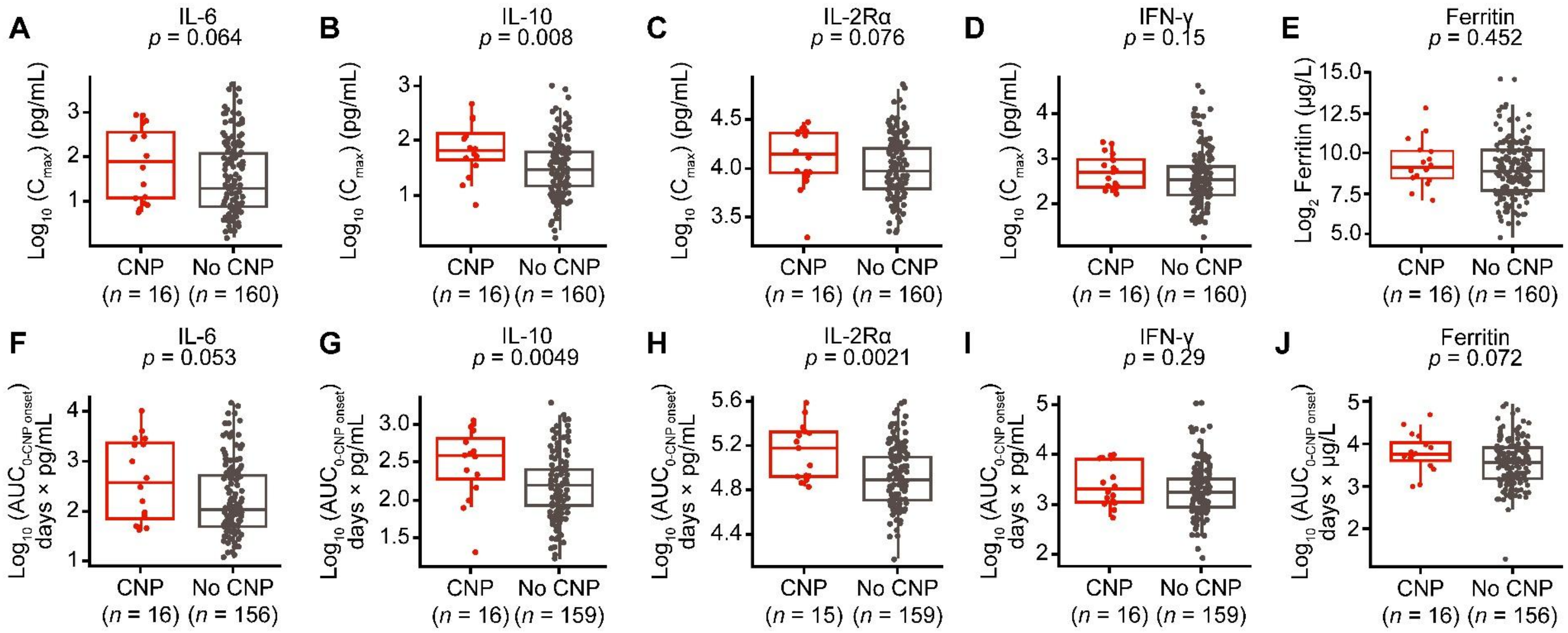

Figure S5. Post-infusion levels of serum inflammatory markers in patients with and without CNP in CARTITUDE-4. C_{max} of (A) IL-6, (B) IL-10, (C) IL-2Rα, (D) IFN-γ, and (E) ferritin. $AUC_{0\text{-CNP onset}}$ of (F) IL-6, (G) IL-10, (H) IL-2Rα, (I) IFN-γ, and (J) ferritin. The median day to CNP onset was used to calculate an equivalent timepoint for patients without CNP. *$AUC_{0\text{-CNP onset}}$* area under the concentration-time curve from time 0 to CNP onset, *C_{max}* maximal level, *CNP* cranial nerve palsy, *IFN* interferon, *IL* interleukin.

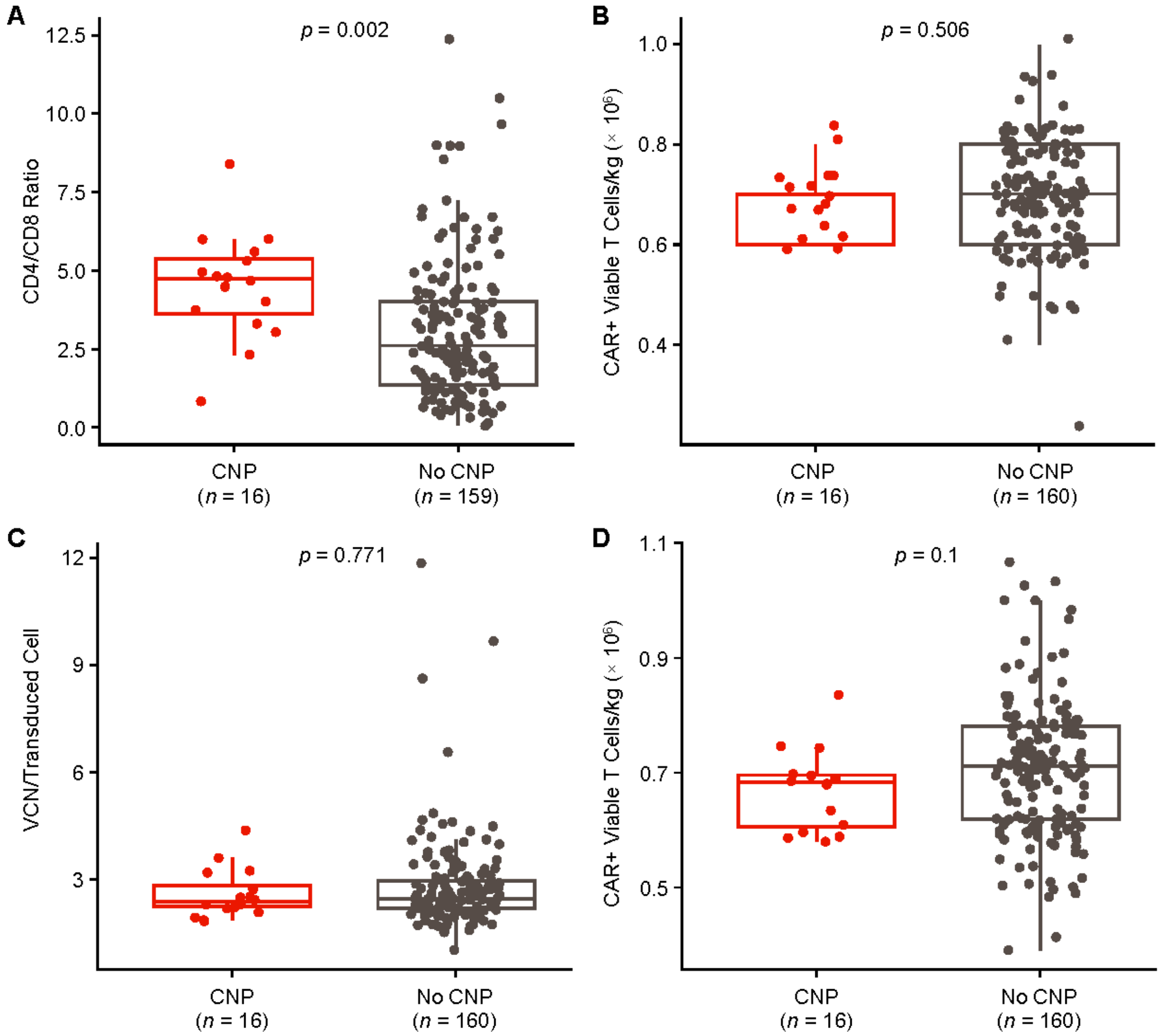
A
p = 0.002
12.5
10.0
7.5
5.0
2.5
0.0
CD4/CD8 Ratio
CNP
(n = 16)
No CNP
(n = 159)
B
p = 0.506
1.0
0.8
0.6
0.4
CAR+ Viable T Cells/kg (× 10⁶)
CNP
(n = 16)
No CNP
(n = 160)
C
p = 0.771
12
9
6
3
VCN/Transduced Cell
CNP
(n = 16)
No CNP
(n = 160)
D
p = 0.1
1.1
0.9
0.7
0.5
CAR+ Viable T Cells/kg (× 10⁶)
CNP
(n = 16)
No CNP
(n = 160)

Figure S6. (A) CD4:CD8 ratio in DP, (B) CAR+ viable T cells/kg in DP, (C) VCN/transduced cell in DP, and (D) CAR+ T-cell dose in patients with and without CNP in CARTITUDE-4. *CAR* chimeric antigen receptor, *CNP* cranial nerve palsy, *DP* drug product, *VCN* vector copy number.

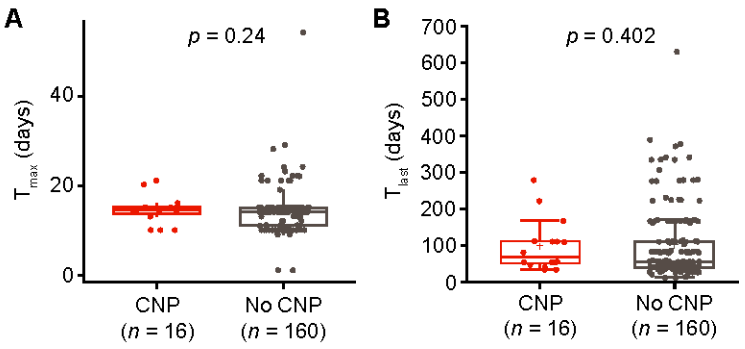

Figure S7. (A) T_{max} and (B) T_{last} in patients with and without CNP in CARTITUDE-4. *CAR* chimeric antigen receptor, *CNP* cranial nerve palsy, T_{last} sampling time of last measurable (i.e., not below quantitation limit) CAR+ T cells (persistence), T_{max} time to peak expansion of CAR+ T cells.

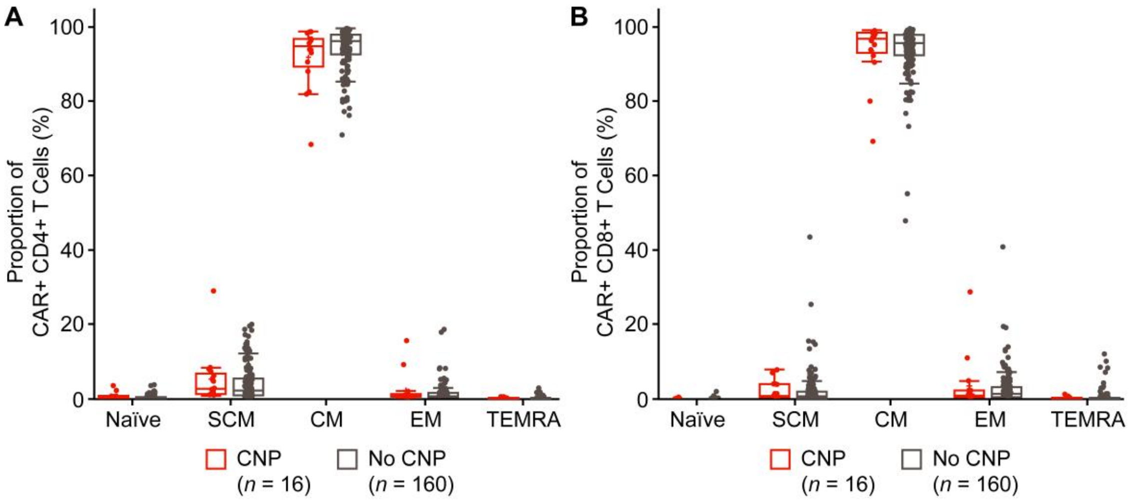

Figure S8. Memory T-cell phenotypes at T_{max} in patients with and without CNP in CARTITUDE 4. (A) CAR+ CD4+ T-cell compartment and (B) CAR+ CD8+ T-cell compartment. *CAR* chimeric antigen receptor, *CM* central memory, *CNP* cranial nerve palsy, *EM* effector memory, *SCM* stem cell memory, *TEMRA* effector memory re-expressing CD45RA T cell, T_{max} time to peak expansion of CAR+ T cells.
